# The impact of artisanal mining on rehabilitation efforts of abandoned mine shafts in Sutherland goldfield, South Africa

**DOI:** 10.4102/jamba.v11i2.688

**Published:** 2019-06-13

**Authors:** Sphiwe E. Mhlongo, Francis Amponsah-Dacosta, Confidence Muzerengi, Wilson M. Gitari, Abuh Momoh

**Affiliations:** 1 Department of Mining and Environmental Geology, University of Venda, Thohoyandou, South Africa; 2 Department of Ecology and Resource Management, University of Venda, Thohoyandou, South Africa; 3 Department of Geology and Mining, University of Jos, Jos, Nigeria

**Keywords:** Artisanal Gold Mining, Abandoned Mine Shafts, Sutherland Goldfield, South Africa

## Abstract

Abandoned gold mine sites are generally characterised by severe environmental problems and physical hazards. Because of socio-economic problems confronting communities around abandoned mine sites, historic and abandoned gold mines have become hot-spots for artisanal and small-scale miners. These mining activities at times thwart the efforts of rehabilitation at these sites. This article details how artisanal mining operations have frustrated rehabilitation efforts of abandoned mine shafts in the Sutherland goldfield. The field investigation of abandoned shafts and analysis of the nature of artisanal mining operations in the Sutherland goldfield revealed that artisanal mining involving digging around collars of sealed shafts is a major threat to the stability of the shafts and their sealing structures. In addition, artisanal mining operations have increased the safety risks of the abandoned shafts in the area. This has also been worsened by the fact that a large number of people, especially women and children, are exposed to the hazards of the abandoned mine sites. This article emphasises an urgent need for the development of holistic and cohesive strategies for dealing with the problems of abandoned gold mine shafts wherever they exist in the country as opposed to simply closing them up.

## Introduction

In South Africa, the term ‘abandoned mines’ refers to those mines for which a closure certificate has not been issued and no party can be traced to assume responsibility for their liabilities. In general, abandoned mines are found in all countries and regions of the world with long histories of mining (Hentschel, Hruschka & Priester 2002). Mining of gold in the Sutherland (Giyani) goldfield began soon after gold was discovered by Sutherland and Button in 1970 (Steenkamp & Clark-Mostert 2012; Word & Wilson 1998). Currently, all the gold mines in the Sutherland goldfield have ceased operating and they were abandoned without any rehabilitation. The known mines that operated in the Sutherland goldfield (formerly known as the Giyani Greenstone Belt) include Klein Letaba, Louse Moore, Fumani, New Union (also known as Golden Osprey), Franke and Birthday. In all these mines, gold mining was carried out by small- to medium-scale underground mining operations. A small portion of the ore body at Franke Mine was mined by shallow open pits of an average depth of 25 m. Part of the pit was linked with underground mine workings; thus, the mine is characterised by a huge, dangerous excavation that is filled with water.

In all regions and/or areas where abandoned mines are found, they turn to be the major source of environmental and physical hazards as well as socio-economic problems. Over the past few years (from around 2008), there has been a continuous work of trying to address the hazards of abandoned mines in the Sutherland (Giyani) goldfield. According to the Department of Mineral Resources (2010), at the end of 2010, open shafts in the Sutherland (Giyani) goldfield and a few asbestos workings were on top of the priority list of abandoned mines rehabilitation in Limpopo province. The main objectives of the abandoned mines rehabilitation work in the Sutherland goldfield are to identify and locate mine shafts and other openings associated with the abandoned mines that pose a threat to public health and safety and to propose solutions for closing the hazardous openings.

Illegal mining has become a major problem and is on the rise in some of the provinces of South Africa. These illicit mining activities in the old, defunct mines have, in the past two years, become a major challenge for the Department of Mineral Resources, mining companies and local authorities (Hutchings & Mkhize 2014). Illegal mining at abandoned mine sites is common in the Sutherland goldfield, where informal miners excavate and scavenge for ore and gold to sell. A major tragedy is looming in abandoned and disused mines if government does not take decisive action to curb thriving illegal mining.

Illegitimate small-scale gold mining activities in Sutherland goldfield are conducted without any planning and are undertaken by workers who do not have the technical know-how. Therefore, excavations dug by these miners are left unmarked, representing a hazard for population and livestock. Areas that have undergone some form of rehabilitation few years ago are greatly disturbed, thereby worsening the environmental degradation of the abandoned mine sites. This article provides comprehensive information on the nature of artisanal mining operations in the Sutherland goldfield and the manner in which these operations have frustrated the rehabilitation efforts of abandoned mine shafts.

### The study area

**FIGURE 1:**
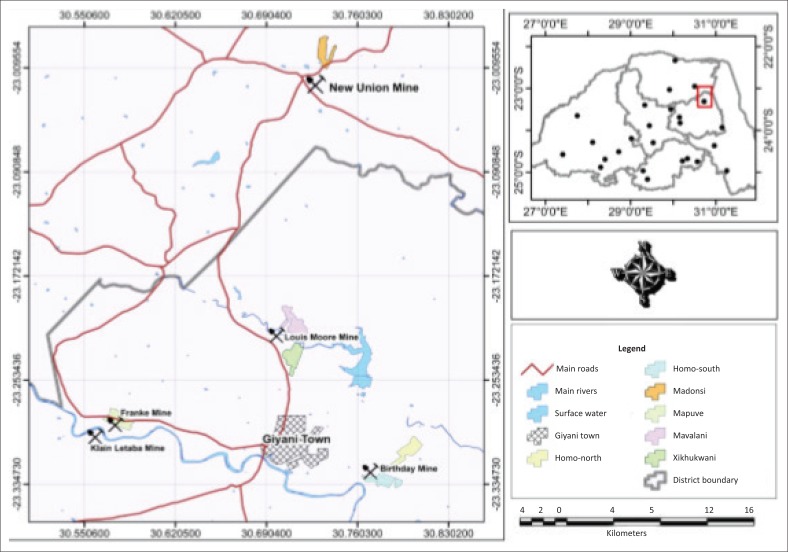
The location of the studied abandoned mines in the Giyani area.

The Sutherland goldfield is a 70-km-long north-easterly trending greenstone sequence found along the north-eastern corner of the Limpopo province of South Africa (Steenkamp & Clark-Mostert 2012). The abandoned mine sites studied in this work include the New Union Mine (found at Ka-Madonsi village), Louis Moore Mine (in the vicinity of Mavalani and Xikhukwani villages), Birthday Mine (at Ka-Homo village), Franke Mine and Klein Letaba Mine (both found around Ka-Mapuve village) (see Figure 1). Although these mines contributed differently to gold production in the Sutherland goldfield, collectively (including Fumani Mine) they account for more than 10 tonnes (97%) of all the gold known to have been recovered from this goldfield (Word & Wilson 1998).

## Methodology

The impacts of artisanal gold mining on the rehabilitation efforts of abandoned shafts were established through carrying out an intensive field characterisation of the abandoned mine shafts in the study area. The method employed in the characterisation of the mine shafts of selected abandoned mines (i.e. New Union, Louis Moore, Birthday, Franke and Klein Letaba) began with locating the mine shafts found around the sites. Because of the fact that the abandoned mine shafts in the Sutherland goldfield do not have structures such as head frames that can be used as an indicator of their presence and location, a systematic traversing around the abandoned mine sites was conducted. This assisted in locating a significant number of abandoned shafts. A handheld geographical positioning system (GPS) was used in marking the location of all the identified mine shafts. A detailed description of the current state of the identified abandoned mine shafts was provided. The description of the shafts placed emphasis on the stability of the shafts sealing structure and the nature of the hazards posed by the shaft to the members of the community as well as to animals. The field analysis of the way and manner in which artisanal gold mining is conducted in the Sutherland goldfield was conducted. The fieldwork also looked at the different ways by which artisanal gold mining operations have affected the shaft closure efforts and the impact of these activities on the environment at large.

## Results

Generally, there is no universal definition of ‘artisanal mining’ (Phiri et al. 2015). According to Hilson (2002), artisanal mining refers to informal and formal activities that are carried out using low technology or minimal machinery. These activities are normally undertaken by individuals and/or small groups that are mostly unskilled and uneducated and from poverty-stricken communities which are mostly rural (Hoedoafia 2014). In South Africa, artisanal gold mining is commonly carried out around abandoned and historic mine sites. The magnitude at which these activities are carried out in South Africa can be seen through the fact that the Gauteng province alone is estimated to be having approximately 30 000 artisanal miners (also referred to as Zama-Zamas), who operate around the abandoned or historic gold mining sites (Capel 2017). The next section provides a detailed description of artisanal gold mining activities and their effects on the rehabilitation efforts of abandoned mine shafts in the Sutherland goldfield.

### Artisanal gold mining in the Sutherland goldfield

**FIGURE 2:**
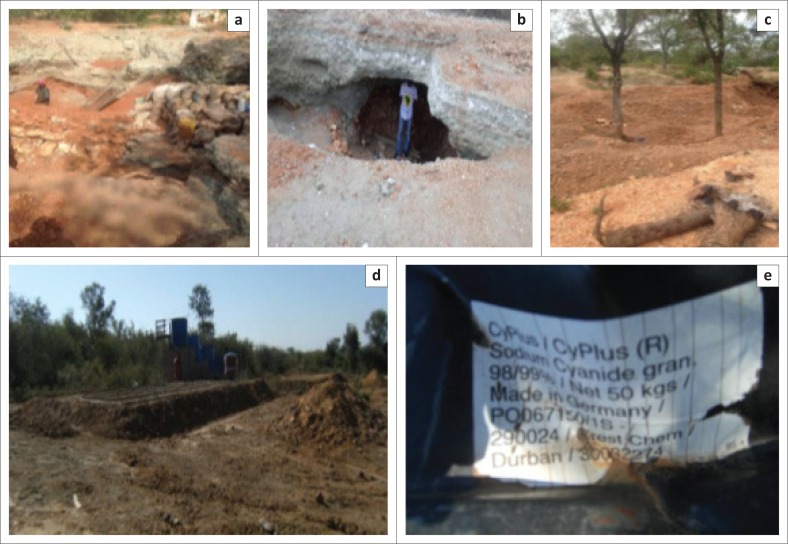
Artisanal mining at the Sutherland goldfield: (a) Fine gravel digging operations, (b) hazardous excavation at Louis Moore Mine, (c) the impact of excavations on trees around New Union Mine, (d) an abandoned cyanide heap leaching setup (e) and an empty container of the leaching chemical used.

Artisanal gold mining in the Sutherland goldfield involves digging and collection of gold-bearing fine sediments from historic or abandoned mine sites. In the collection of sediments, the artisanal miners target abandoned mining sites, especially the abandoned shafts, mineral processing areas and dilapidated buildings. They dig shallow pits below the foundations of abandoned concrete structures, including the shaft collars. The excavated material is sieved in shallow pits (as shown in Figure 2a) to ensure that only sand-size material is collected, whilst gavel and boulders are dumped outside the pits as waste. In some cases, excavations that are approximately 2 m deep have taken a form of underground adits at about 3 m below the surface (see Figure 2b). This puts in risk the lives of both the miners and children who mostly play around the operation site as their parents and/or guardians are involved in excavation and material collection activities. Dangerous diggings were also identified under sealed shaft collars and below high structures, such as silos and tall walls of abandoned buildings. These have contributed significantly to the instability of the shafts sealing structures as well as the dilapidated buildings and mineral processing plant infrastructure (those are silos and concrete mounting structures).

The diggings have affected the vegetation growth in the areas where artisanal gold mining happens around the abandoned mine sites. This is because of the fact that the operation of sediments collection has led to cutting and uprooting of trees that have over the years grown in the abandoned mine sites (see Figure 2c).

The material excavated from the mining sites is packed in 12.5 kg sacks and transported to washing sites built away from the mining site. The washing or sluicing tables are built with slopes of about 10°–15° along the surface water sources (Steenkamp & Clark-Mostert 2012) and in a manner that allows easy circulation of washing water. The concentrate obtained from sluicing operations is sold without further processing. Generally, it is a very small amount of material that gets to be washed before it is sold. Most artisanal miners prefer to sell the material from mining sites without washing or getting the concentrate from the material. During the visit to the abandoned mine lands around the Giyani area in March 2014, about 20 bags (yielding 1 g processed gold) of sediments collected from Louis Moore Mine were being sold at a price of R350.

In addition, the artisanal miners at New Union Mine attempted extracting gold from the mine tailings using cyanide heap leaching method. In this method, the tailings material was piled up to 0.5 m onto the impermeable floor (cemented floor). The stacked material, shown in Figure 2d, was then sprayed with sodium cyanide solution. As the processing solution (cyanide solution) peculates into the heaped tailing material, it liberates and mobilises the remnants of gold into the solution to form an impregnated solution. The solution containing gold was being collected through cemented (impermeable) solution channels into solution tanks buried in the ground.

### Rehabilitation of abandoned mine shafts

**FIGURE 3:**
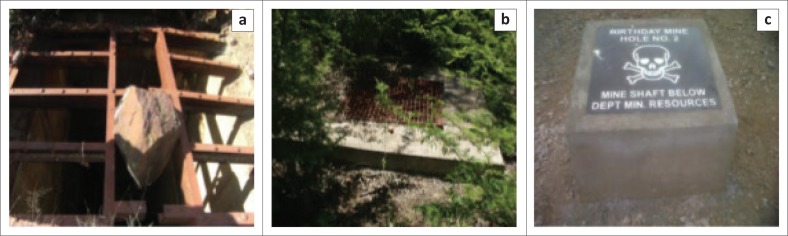
Shaft-closing structures at Sutherland goldfield: (a) heavy steel grate, (b) steel wire screen and concrete and (c) concrete plugs with landmark.

There are three shaft closing strategies that were used in addressing the safety concerns of abandoned shafts in the Sutherland goldfield. The main focus of shaft closing operations in the area was to reduce or eliminate the physical hazards associated with the abandoned mine shafts. The strategy employed earlier in closing open shafts was placing a steel grate (see Figure 3a) on the shaft in order to prevent both human and animals from falling into the shafts. All the mine shafts that were previously closed but now opened at New Union Mine were closed using this mine shaft closing technique. There was no evidence of the use of steel grate closure in other mines. In general, the steel grate mine closing structure had a disadvantage of being easily removed by artisanal or illegal miners who wanted to gain access to underground workings with the purpose of mining the remnants of the deposit. Moreover, the steel used to build this shaft closing structures attracted the members of the community to destroy the structure, thus leaving the shafts open again.

The second shaft closing approach involved completely and/or partial sealing of the shaft with reinforced concrete applied concurrently with steel grate, and in some cases it was applied with steel wire screen closure (see Figure 3b). Shafts closed in this manner were easily opened by artisanal miners by simply removing the steel wire screen or destroying the concrete structure. In some cases, artisanal miners mined the material below the concrete shaft collars, thus removing the foundation of the shaft closing structure and that of the entire shaft collar. The evidence of the use of this shaft closing strategy was identified in four of the studied abandoned mine sites, namely, Louis Moore, Franke, Birthday and Klain Letaba.

The currently implemented strategy involves the installation of a massive concrete plug at about 3 m – 10 m below the surface of the earth. According to the Department of Mineral Resources (2010), the concrete plugs do not contain any metallic reinforcing materials that may attract community members to destroy the plugs in search of these materials. The plugs are completely buried under the ground, with only about 2.5 tonnes of concrete landmark being exposed on the surface (see Figure 3c). In all mine shafts where the concrete plug closing approach was used, they have not been affected by artisanal mining operations. However, cases of theft of granite slabs placed on landmarks were reported. This shaft closure option has so far been only used in closing mine shafts at Birthday Mine. Table 1 presents the summary of the current state of all the identified mine shafts around the abandoned mine sites in the Sutherland goldfield.

**TABLE 1:** The current state of the mine shafts at Sutherland goldfield.

Mine Site	Shaft code	Description		Location	
		State of mine shaft	State of closing structure	Longitude	Latitude
New Union Mine	NUM-1	^a^Open	-	23° 01’ 2”	30° 43’ 37”
	NUM-2	^a^Open	-	23° 01’ 8”	30° 43’ 38”
	NUM-3	^b^Opened	Completely removed	23° 01’ 28”	30° 43’ 31”
	NUM-4	^a^Open	-	23° 01’ 8”	30° 44’ 7”
	NUM-5	^a^Open	-	23° 01’ 12”	30° 44’ 6”
	NUM-6	^a^Open	-	23° 01’ 16”	30° 44’ 45”
	NUM-7	^b^Opened	Completely removed	23° 01’ 21”	30° 44’ 3”
Louis Moore Mine	LMM-1	^c^Opened	Destroyed and not stable	23° 13’ 13”	30° 41’ 48”
	LMM-2	^c^Opened	Destroyed and not stable	23° 13’ 13”	30° 41’ 45”
Franke Gold Mine	FGM-1	^c^Opened	Destroyed but still stable	23° 17’ 21”	30° 24’ 17”
	FGM-2	^d^Sealed	Old stable structure	23° 17’ 20”	30° 34’ 16”
	FGM-3	^d^Sealed	Old stable structure	23° 17’ 19”	30° 34’ 17”
	FGM-4	^d^Sealed	Old structure is stable	23° 17’ 22”	30° 34’ 5”
	FGM-5	^d^Sealed	Old structure is stable	23° 17’ 35”	30° 34’ 38”
Birthday mine	BDM-1	^d^Sealed	Stable new structure	23° 19’ 23”	30° 46’ 19”
	BDM-2	^d^Sealed	Stable new structure	23° 19’ 22”	30° 46’ 19”
	BDM-3	^d^Sealed	Stable new structure	23° 19’ 22”	30° 46’ 20”
	BDM-4	^d^Sealed	Stable new structure	23° 19’ 24”	30° 46’ 17”
	BDM-5	^d^Sealed	Stable new structure	23° 19’ 29”	30° 46’ 14”
	BDM-6	^d^Sealed	Stable new structure	23° 19’ 28”	30° 46’ 14”
	BDM-7	^d^Sealed	Stable new structure	23° 19’ 38”	30° 46’ 16”
	BDM-8	^d^Sealed	Stable new structure	23° 19 39”	30° 46’ 16”
	BDM-9	^d^Sealed	Stable new structure	23° 19’ 38”	30° 46’ 18”
	BDM-10	^d^Sealed	Old stable structure	23° 19’ 35”	30° 46’ 15”
Klein Letaba Mine	KLM-1	^d^Sealed	Old stable structure	23° 18’ 9”	30° 44’ 57”

a , The shafts were never closed.

b , The shaft sealing structure has been completely removed, thus leaving the shaft open.

c , Part of the shaft is opened.

d , Completely closed mine shafts.

## Discussion

Mining is one of the main economic activities in all developing countries like South Africa. The gold mining industry in South Africa is well established and it is dominated by large-scale mining operations. However, there is still a significant amount of gold that is mined by artisanal and small-scale miners. These are groups of people who mine using rudimentary tools in labour-intensive operations and are without mining rights. According to Hinton, Veiga and Veiga (2003), artisanal mining is an accentual activity in developing countries as it provides an important source of livelihood. Artisanal and/or illegal gold mining is said to be responsible for a yearly loss of about R5.6 billion to the South African gold mining revenue (Moodley 2013).

**FIGURE 4:**
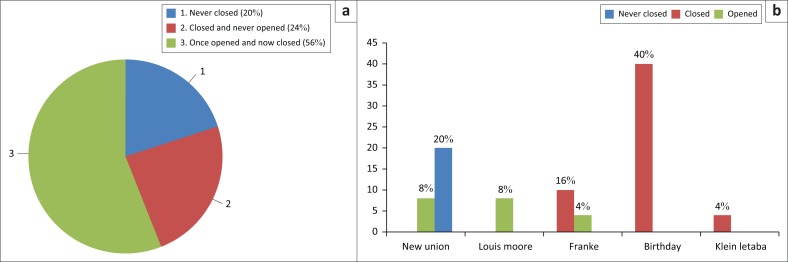
(a) The impact of artisanal mining on the rehabilitation efforts of abandoned mine shafts and (b) state of mine shafts in the five abandoned mines in the Sutherland goldfield.

According to Hinton et al. (2003), throughout the world, artisanal mining communities vary from culture to culture, region to region, time to time and from one mine to another, and South Africa is no exception. Artisanal gold mining in South Africa varies from one region to another and from one mine to another. Artisanal miners generally mine using various interchangeable methods, such as shallow open pits, riverbank excavation and bed panning (Bhebhe et al. 2013). In the vicinity of the City of Johannesburg, artisanal (illegal) miners broke concrete slabs that covered abandoned mine shafts to gain access to old mine workings where they could mine, cook and sleep for weeks before they come to the surface (Crowley 2014). In the Sutherland goldfield, the artisanal gold miners excavated shallow pits around the abandoned gold mines and destroyed concrete slabs used in closing the shafts. About 56% of all the studied mine shafts were found to have been once opened by artisanal miners in the Sutherland goldfield, whilst 24% shafts were closed ones and never opened. It was only 20% of the studied mine shafts that were found to have never been closed since the time of abandonment of the mines (see Figure 4a). All mine shafts that were never closed were identified around New Union Mine, whilst most of the closed shafts were identified at Birthday Mine, which was also the mine that was found to be less affected by artisanal gold mining activities. It is also worth mentioning that although the mine shafts at Birthday Mine were found closed (see Figure 4b), several inappropriate strategies were used to close these shafts before, but all failed. The evidence of destruction of the shafts closing structures was identified in all the studied abandoned mine sites except Klein Letaba Mine.

Results of the field studies have shown that artisanal gold mining operations in the Sutherland goldfield have worsened both the physical hazards and environmental impacts of the mining activities at the abandoned mine sites. The practice of opening up closed shafts or mining the shaft walls has exposed the miners to the risk of falling into underground mine workings, which, according to Parnham (n.d.), are approximately 250 m deep. Although there are no reported cases of injuries or fatalities of artisanal miners because of the fall into abandoned underground mines at the Sutherland goldfield, such cases are known in other goldfields in the country (Hartnady 2009). Besides getting into the underground mine workings, the safety of the artisanal mining community is threated by these operations that involve digging and developing unstable shallow underground excavations.

Artisanal gold mining in the Sutherland goldfield was also identified to be associated with environmental problems that are mainly caused by irresponsible digging of shallow pits throughout the abandoned mine sites, use of sodium cyanide in leaching gold from old mine tailings and the construction of sluicing tables along the river banks. The cutting and uprooting of trees and the random excavation of shallow pits and dumping of waste (gravel material generated from sieving of gold-bearing sediments or fines) have caused considerable damage to the landscape. The land degradation caused by artisanal gold mining in the Sutherland goldfield is of great concern as it is characterised by a significant change in the landscape, biodiversity and topsoil structure in all areas of artisanal gold mining. These problems were also identified by researchers, such as Kitula (2006) and Ingram et al. (2011), to be common in areas of artisanal gold mining throughout the world. The use of cyanide heap leaching method is also a major threat to the environment and it has potential health impacts on the members of the community. Although this method is considered cost-effective in processing low-grade ores (Eisler & Wiemeyer 2004; EPA 1994), it has the disadvantages of leaving behind a huge amount of toxic waste. In addition, if the processed solution is allowed to spill out of the circuit, the environmental problems of the process are certain and they can have severe health impacts on people and animals in the area or region (Reinhardt 2008). For example, during the 1980s, cyanide from leaching ponds in California, Nevada and Arizona killed about 7613 animals (Eisler 1991). The construction of sluicing tables along the river systems has affected the surface water systems. According to Steenkamp and Clark-Mostert (2012), rehabilitation of the damages that artisanal or small-scale miners have caused to the river systems in the Sutherland goldfield will require considerable effort and resources to remedy the situation.

## Conclusion

This article revealed that there is an urgent need for development and implementation of abandoned mines rehabilitation strategies that will put an end to both physical and environmental hazards whilst addressing the socio-economic issues left by the unplanned closure of these mines. The strategies to be implemented in the rehabilitation of abandoned mines or mine features should be those that open new economic opportunities for the host communities.

The socio-economic issues that exist within the communities around the abandoned mines in the Sutherland goldfield have forced many people to embark on artisanal gold mining ventures. These mining activities have in one way or another reversed the efforts of cleaning up the hazards of the abandoned mine shafts. It was discovered that more than half (56%) of the shafts were closed at some point in time, but were later opened by artisanal miners. These nefarious small-scale mining activities have undoubtedly thwarted the ongoing rehabilitation efforts. Resources that could have been used in closing shafts at other mines, where the majority of shafts were never closed, are still being utilised in closing shafts that were once rehabilitated, such as New Union Mine.

Artisanal mining operations have exacerbated the environmental problems and public safety and health threats. By their very nature, artisanal gold mining operations in the Sutherland goldfield expose a large number of people, most of whom are women and children, to the safety hazards of the abandoned mines. Digging and washing of gold-bearing sediments around the abandoned mine sites and surface water bodies (rivers) have shown negative effects on the environment. Mine waste (cyanide-contaminated soils and empty sodium cyanide containers) at the abandoned mine sites poses serious threat to human and other aspects of the environment. There is a risk that some members of the public are collecting and using pipes and solution tanks previously used in cyanide leaching for domestic purposes. Moreover, there is a risk of the cyanide-contaminated soils being eroded to the nearby rivers, and this may have impact on the aquatic life.
